# The Changing Landscape in the Genetic Etiology of Human Tooth Agenesis

**DOI:** 10.3390/genes9050255

**Published:** 2018-05-16

**Authors:** Meredith A. Williams, Ariadne Letra

**Affiliations:** 1 University of Texas Health Science Center at Houston School of Dentistry, Houston, TX 77054, USA; Meredith.A.Whitman@uth.tmc.edu; 2 Department of Diagnostic and Biomedical Sciences, University of Texas Health Science Center at Houston School of Dentistry, Houston, TX 77054, USA; 3 Center for Craniofacial Research, University of Texas Health Science Center at Houston School of Dentistry, Houston, TX 77054, USA; 4 Pediatric Research Center, University of Texas Health Science Center at Houston McGovern Medical School, Houston, TX 77030, USA

**Keywords:** tooth agenesis, etiology, gene, inheritance

## Abstract

Despite much progress in understanding the genetics of syndromic tooth agenesis (TA), the causes of the most common, isolated TA remain elusive. Recent studies have identified novel genes and variants contributing to the etiology of TA, and revealed new pathways in which tooth development genes belong. Further, the use of new research approaches including next-generation sequencing has provided increased evidence supporting an oligogenic inheritance model for TA, and may explain the phenotypic variability of the condition. In this review, we present current knowledge about the genetic mechanisms underlying syndromic and isolated TA in humans, and highlight the value of incorporating next-generation sequencing approaches to identify causative and/or modifier genes that contribute to the etiology of TA.

## 1. Introduction

Tooth development requires a sequential and reciprocal series of signaling interactions between the oral epithelium and the neural crest-derived mesenchyme, which are under strict genetic control by a number of signaling molecules and their downstream signaling pathways. During these stages, the continuous interplay of inductive signals between epithelia and mesenchyme in a precisely organized manner results in the formation of distinct and highly specialized structures, such as incisor, canine, premolar and molar teeth [1]. Disturbances during any step of tooth development may affect growth, differentiation and pattern formation [2].

Tooth agenesis (TA) is the congenital absence of one or more permanent teeth, and results from disturbances during early stages of tooth development. TA is recognized as the most common abnormality of dental development and may appear as a characteristic feature of ≈150 syndromes, although it is more frequent as an isolated trait that may appear sporadically or segregate in families [3,4]. Autosomal dominant, autosomal recessive, or X-linked patterns of inheritance have been described for TA, with considerable variation in penetrance and expressivity. Based on the number of missing teeth other than third molars, TA is referred to as hypodontia (≤5 teeth missing) or oligodontia (≥6 teeth missing). Anodontia refers to the absence of all permanent teeth and is associated with syndromic forms of TA [5].

There are large differences in the prevalence of TA, whether it refers to hypodontia or oligodontia, and also among genders and different racial populations. In general, population studies have revealed that the prevalence of isolated hypodontia ranges from 3–10%, while oligodontia is less common with a reported prevalence of 0.1–0.5%, excluding third molars. Upon inclusion of third molars, the prevalence rises to 25% [4,6]. The most commonly missing teeth are permanent mandibular second premolars, maxillary lateral incisors, and maxillary second premolars [3]. Females are more affected than males in a 3:2 ratio [5].

TA may be occasionally caused by exogenous factors, such as infections, trauma, chemotherapy, or radiotherapy, however, the majority of cases are due to genetic factors [7]. Studies of odontogenesis in mice have elegantly shown that tooth development is under strict genetic control, which determines the positions, numbers and shapes of different teeth [8]. Over many years, studies using transgenic animals provided functional data showing that disruption of genes in the bone morphogenetic protein (BMP), fibroblast growth factor (FGF), sonic hedgehog (SHH), and wingless-related integration site (WNT) signaling pathways resulted in severe abnormalities of tooth development ranging from defects in tooth patterning to complete arrest of tooth development [1,4].

In humans, mutations in several genes have been reported as causal for TA. However, known mutations explain a restricted number of cases and much remains to be learned about the underlying molecular mechanisms in TA [4]. Epigenetic regulation affecting a network of interconnected signaling pathways that included tooth development was also suggested to play a role in the etiology of TA [9]. Recently, new technologies such as next-generation sequencing have proven to be valuable tools in the identification of novel TA genes and variants, and begun to elucidate the genetic defects responsible for this condition [10,11].

The purpose of this review is to present an overview of genes and pathways identified as having a role in human TA, and discuss recent findings from next-generation sequencing studies. We also present new perspectives on the potential molecular mechanisms underlying human TA that may bring new possibilities for future prevention and treatment.

## 2. Genetic Basis of Tooth Agenesis

### 2.1. Syndromes Associated with Tooth Agenesis

More than 150 syndromes include TA as a phenotype. These include mostly oral-facial cleft syndromes and ectodermal dysplasia syndromes [12,13]. Teeth share developmental mechanisms with other ectodermal organs in terms of timing and the cellular processes involved, therefore genes involved in odontogenesis may play a role in other developmental processes [12,14]. A summary of the syndromic forms of TA and their etiologic genes is presented in Table 1.

#### 2.1.1. Ectodermal Dysplasia Syndromes

Ectodermal dysplasias (ED) comprise a group of disorders characterized by a combination of findings involving defects in the skin, hair, nails, teeth, exocrine and sebaceous glands. Distinct types of ED have been reported, caused by mutations in different genes. X-linked recessive hypohidrotic (HED) is the most common ED and caused by mutations in the *EDA* (ectodysplasin A) gene. Currently, more than 200 mutations in *EDA* have been found in individuals with HED. The EDA protein is a type-II trimeric transmembrane protein belonging to the tumor necrosis factor (TNF) ligand superfamily which functions as a signaling molecule during epithelial morphogenesis [98]. Upon stimulation, EDA binds to its receptor, EDAR (ectodysplasin A receptor), which in turn binds to the adaptor EDARADD (EDAR-associated death domain) for activation of downstream target proteins. Mutations in *EDAR* and *EDARADD* cause autosomal dominant and recessive forms of HED [4,7]. In addition to variable involvement of teeth in distinct forms of ED, minor ectodermal anomalies are often observed.

Witkop syndrome is a rare autosomal dominant ED involving the teeth and nails, caused by mutations in the *MSX1* (muscle segment homeobox, homolog 1) gene. Although a few reported cases have sparse or fine hair, most affected individuals have normal hair and sweat glands [99]. *MSX1* is a homeobox gene that belongs to a family of transcription factors that are expressed in overlapping patterns at multiple sites of tissue interactions during vertebrate development [2]. In mice, *MSX1* is essential for initiation of the tooth germ during the bud stage and then promotes the odontogenic potential of the dental mesenchyme [2]. Mice lacking *MSX1* protein function present cleft palate, deficient mandibular and maxillary alveolar bones, and arrest of tooth development [100]. Further, *MSX1* is a direct downstream target of WNT/ß-catenin signaling during craniofacial development and regulates the expression of additional genes within this pathway, including *BMP2* (bone morphogenetic protein 2) and *BMP4* (bone morphogenetic protein 4) [4]. Over 20 *MSX1* mutations have been reported in association with both syndromic and nonsyndromic TA phenotypes in humans.

Odontoonychodermal dysplasia (OODD) and Schopf–Schulz–Passarge syndrome (SSPS) are autosomal recessive disorders caused by homozygous or compound heterozygous missense mutations in *WNT10A* (wingless-type MMTV integration site family, member 10A). Affected individuals have dry hair, severe TA, smooth tongue, palmoplantar keratosis, and dystrophic nails [93,94]. *WNT10A* is thought to function through the canonical Wnt-ß catenin signaling pathway and activates target genes in the nucleus through binding to *LEF1* transcription factor [101]. During tooth development, continuous Wnt-β catenin signaling in the dental epithelium and mesenchyme is required for tooth formation and morphogenesis [102]. During mouse tooth development, *WNT10A* is first detected in the enamel knot and then shifting to the secondary enamel knot to the underlying mesenchyme and developing odontoblasts [103,104]. In *WNT10A* null mice, however, supernumerary teeth and altered molar crown morphology are observed, in contrast to the TA phenotype in humans [105]. Numerous reports have shown the involvement of *WNT10A* mutations in OODD and SSPS, and in a wide spectrum of autosomal recessive ectodermal dysplasias [93,94,106,107] as well as in isolated TA cases [11,108,109,110].

A homozygous missense mutation (c.626T > C, p.Phe209Ser) in *KREMEN1* (kringle containing transmembrane protein 1), a negative regulator of the Wnt pathway, was identified in four consanguineous Palestinian families with ED. This variant in its heterozygous state was also identified in 6 out of 39 unaffected control individuals [64]. Recently, two additional variants in *KREMEN1* (c.146C > G and c.773_778del) were identified as pathogenic in two Turkish families with suspected ED [11]. *KREMEN1* encodes a kringle domain-containing transmembrane protein that binds to *DKK1*, creating a *DKK1*-Kremen-*LRP6* ligand-receptor complex critical for Wnt signaling [111]. While in this complex, Kremen triggers the internalization of *LRP6* inhibiting Wnt signaling. In the absence of *DKK1*, however, Kremen can increase Wnt signaling through *LRP6* binding. Targeted disruption of the Wnt regulator Kremen in mice induces limb defects and high bone density but no other obvious phenotypes [111].

#### 2.1.2. Oral-Facial Cleft Syndromes

Van der Woude syndrome (VWS) is an autosomal dominant disorder and one of the most common clefting syndromes. Affected individuals present with cleft lip with or without cleft palate (CL/P) and a range of associated features including lower lip pits and TA, which is present in ≈70% of the cases. VWS is caused by mutations in the *IRF6* (interferon regulatory factor 6) gene that encodes a transcription factor highly expressed during craniofacial development and a regulator of keratinocyte proliferation and differentiation [57]. Mice deficient for *IRF6* have abnormal skin, limb and craniofacial development, resultant from a primary defect in keratinocyte differentiation and proliferation. Furthermore, mice homozygous for the *IRF6* null allele have a cleft palate which seems to be caused by a defect in elevation, either as a primary defect or secondary to crowding of the craniofacial structures owing to the constrictive action of the skin or oral adhesions [112]. *IRF6* mutations are recognized as primary genetic causes of isolated and syndromic CL/P [58,113,114].

Cleft lip/palate-ectodermal dysplasia syndrome is a rare, autosomal recessive disorder caused by homozygous loss-of-function mutations of the *PVRL1* (poliovirus receptor-like 1) gene encoding nectin-1 [79]. Nectin-1 is a cell–cell adhesion molecule that is important for the initial step in the formation of adherens junctions and tight junctions; it is expressed in keratinocytes, neurons, and the developing face and palate. Clinical manifestations comprise a unique facial appearance with cleft lip/palate, ectodermal dysplasia, cutaneous syndactyly of the fingers and/or toes, hypodontia and in some cases, mental retardation [80].

#### 2.1.3. Axenfeld–Rieger Syndrome

Axenfeld–Rieger syndrome (MIM #180500) is an autosomal dominant disorder characterized by abnormal development of the anterior segment of the eye, and results in blindness from glaucoma in approximately 50% of affected individuals [115]. Systemic anomalies are associated and include failure of involution of the umbilicus, hypospadias and dental anomalies ranging from microdontia, TA, and tooth malformations [116]. The cause of Axenfeld–Rieger syndrome has been attributed to mutations in the *PITX2* (paired like homeodomain 2) gene, which encodes the earliest transcription marker of tooth development and is expressed in the oral epithelium and dental placode [117]. *PITX2* null mice have tooth development arrested at E12.5 [118]. Additionally, *PITX2* has been shown to play a critical role in establishing left–right asymmetry in vertebrates [119].

#### 2.1.4. Familial Adenomatous Polyposis Syndrome 

Familial adenomatous polyposis (FAP) is an autosomal dominant condition characterized by the development of multiple adenomatous polyps in the colon and rectum with high risk of subsequent malignant transformation. In addition, extracolonic changes occur in many affected subjects. These include epidermoid cysts, desmoid tumours, congenital hypertrophy of retinal pigment epithelium (CHRPE), osseous changes in the jaws and skeleton, and dental anomalies. FAP results from germline mutations in the *APC* (adenomatous polyposis coli) gene on chromosome 5q21 [120]. Approximately 17% of individuals with *APC* gene mutations have dental anomalies, particularly supernumerary teeth and compound odontomas, although cases of TA as well as impacted teeth have also been reported. Importantly, in addition to the established association between certain dental anomalies and FAP, an association between TA and genetic predisposition to colon cancer was suggested [22,121,122].

#### 2.1.5. Oligodontia–Colorectal Cancer Syndrome

A germline nonsense mutation in *AXIN2* (c.1996C > T, p.Arg656*) was identified as the likely cause of autosomal dominant oligodontia (severe TA) and colorectal cancer segregating in a large, four-generation Finnish family. Eleven members of the family lacked at least eight permanent teeth, two of whom developed only three permanent teeth. Colorectal cancer or precancerous lesions of variable types were found in eight of the patients with oligodontia [22]. In this same study, a second frameshift mutation leading to a heterozygous 1-bp insertion (c.1994_1995insG, p.706*) in exon 7 of the *AXIN2* gene was identified in an unrelated individual with oligodontia, suggesting that this gene may contribute to both tooth development and tumor development later in life [22]. A recent study showed that variations in colorectal cancer-related genes (*ATF1 and DUSP10*) were significantly associated with TA (albeit in isolated cases); further, Atf1 and Dusp10 expression was detected in the mouse developing teeth from early bud stages to the formation of the complete tooth, suggesting a potential role for these genes and their encoded proteins in toothdevelopment [123]. Taken together, these findings continue to support a potential overlap in the molecular etiology of TA and colorectal cancer, although no cause-effect relationship can yet be established and more research is warranted in this area.

### 2.2. Isolated (Nonsyndromic) Tooth Agenesis

Syndromic forms of TA and genes implicated during normal tooth development in animal models have provided important clues to identify candidate genes for isolated TA in humans. To date, numerous genes have been proposed as etiologic for isolated TA (Table 2).

#### 2.2.1. *MSX1*

**Figure 1:**
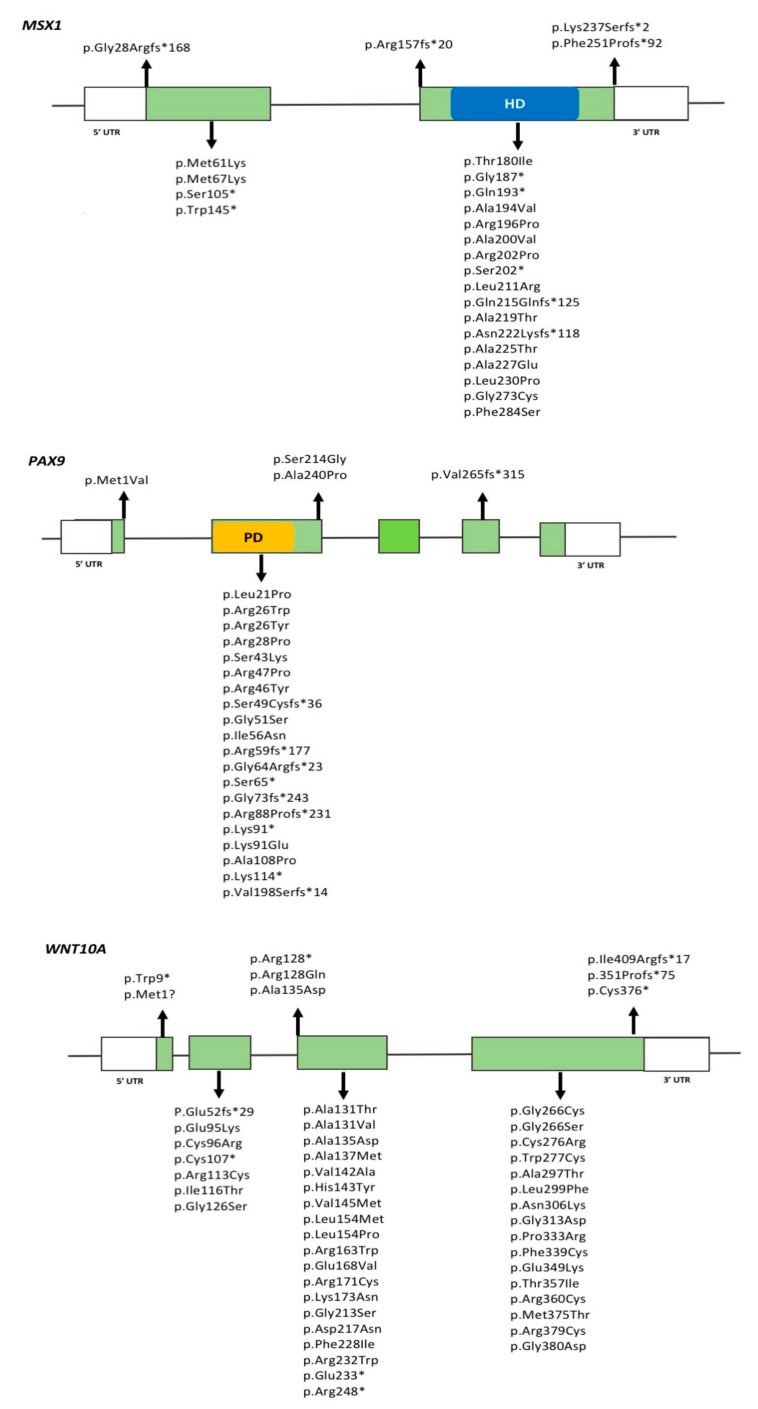
Location of predicted missense, frameshift, and nonsense mutations in *MSX1, PAX9*, and *WNT10A* genes. Green boxes represent exons, horizontal lines between exons represent introns. HD, corresponds to homeodomain in *MSX1*. PD, corresponds to paired domain in *PAX9*. UTR: Untranslated region.

Mutations in *MSX1* were the first to be described in individuals with isolated TA [152]. Since then, over 20 mutations in *MSX1* have been reported in association with isolated TA, most of which are nonsense or missense mutations located in the homeobox domain, and which suggest that haploinsufficiency of *MSX1* underlies TA phenotypes [153] (Figure 1). Mutations in the homeobox domain disrupt DNA-binding and preferentially cause isolated TA, meanwhile variants in the natively unfolded N-terminal part of the protein generally cause oral-facial clefts. These observations suggest that the effect of *MSX1* mutations are directly related to the affected protein domain. *MSX1*-associated TA typically includes missing maxillary and mandibular second premolars and maxillary first premolars.

#### 2.2.2. *PAX9*

*PAX9* belongs to the paired box (PAX) family of transcription factors that are essential for normal development in several multicellular organisms. In addition to *MSX1*, *PAX9* has also long been implicated in isolated TA phenotypes and is one of the most widely studied genes in odontogenesis [4]. *PAX9* is expressed in the presumptive dental mesenchyme to activate signals and initiate tooth development. Absence of *PAX9* in mice results in arrest of tooth development at the bud stage [154,155].

To date, more than 30 variations in *PAX9* have been described in association with TA, most of which are insertions/deletions or missense mutations located in exon 2 of the gene and affecting the paired domain of the PAX9 protein [156] (Figure 1). The presence of *PAX9* variation has primarily been associated with agenesis of permanent second molars, followed by second premolars; a few reports of agenesis of anterior teeth also exist [7,156]. In general, the severity of the TA phenotype is associated with the type of mutation and its impact on *PAX9* function. Individuals with nonsense/frameshift mutations present with a more severe phenotype when compared to those with missense mutations. In TA, known *PAX9* mutations are heterozygous and show autosomal dominant inheritance, indicating that haploinsufficiency is likely contributing to the phenotype. Smaller tooth crown dimensions throughout the dentition have also been reported in TA patients with *PAX9* mutations [157].

#### 2.2.3. *AXIN2*

Rare and common variants in *AXIN2* have been found in association with isolated TA, presenting a mixed pattern of affected teeth [98,124,125]. Agenesis of molars, lower incisors and upper lateral incisors have been described in TA individuals, and the absence of at least one incisor is frequently reported [4,98]. Five *AXIN2* mutations have been widely reported in the literature, including four missense (c.956 + 16A > G; p.Pro50Ser, c.2051C > T; p.Ala684Val, c.2062C > T; p.Leu688Leu, and c.2272G > A; p.Ala758Thr), and one frameshift (c.1994insG; p.Asn666GlyfsX41). The presence of this frameshift mutation was associated with more missing teeth than missense mutations in all affected individuals [98,158].

#### 2.2.4. *WNT10A*

*WNT10A* has been the focus of many genetic studies of TA. Over 50 heterozygous, homozygous as well as compound heterozygous variants in *WNT10A* have been identified in 15.8% of TA patients with 1 to 3 missing teeth, and in ≈52% of patients with >4 missing teeth [109]. Recent genotype–phenotype correlations have provided insights for the role of *WNT10A* in TA. Overall, *WNT10A* compound heterozygous mutations have been found in association with severe TA and a larger number of missing teeth in comparison to individuals with a single variant [108,109,110,148]. While there are no preferential patterns of missing teeth in individuals with *WNT10A* variants, the absence of maxillary and mandibular molars, as well as mandibular incisors is often reported [149]. Of note, heterozygous *WNT10A* variants have also been identified in unaffected individuals in TA families, as well as in unrelated control individuals with no TA or family history of TA [108,109]. It has been estimated that approximately 41% of individuals showing a single heterozygous variant in *WNT10A* will not have TA [148].

Figure 1 shows *WNT10A* variants identified in TA patients. A few *WNT10A* variants have been suggested to be common ‘hotspots’ for mutations in specific populations. For example, the c.637G > A (p.Gly213Ser) variant has been found more frequently in Asian populations [108,149], meanwhile the c.682T > A (p.Phe228Ile) variant has been widely reported in homozygous or heterozygous forms in Caucasian individuals with TA, but also in normal controls at a frequency of 2.3% [109]. The Phe228Ile variant is the most commonly found variant, and often described in combination with additional variants in *WNT10A* or in other genes [159,160]. These findings support an oligogenic inheritance model for TA as discussed later in this review.

#### 2.2.5. *LRP6*

*LRP6* (LDL receptor related protein 6) is a co-receptor in the Wnt/β-catenin pathway and has been recently reported to contribute to isolated TA in different studies [11,136,137]. Six variants, including a nonsense variant (c.1779dupT, p.Glu594*), two insertion (c.2224_2225dupTT, p.Leu742Phefs*7 and c.1144_1145dupAG, p.Ala383Glyfs*8) and a splice-site (c.3607 + 3_6del, p.?) variant resulting in a truncated mRNA product, as well as a missense variant (c.56C > T, p.Ala19Val) were found in individuals with sporadic TA and/or segregating with TA in families [11,136,137]. In mice, *LRP6* expression was noted in the tooth follicle and inner enamel epithelium [137], while homozygous deletion of *LRP6* led to severe skeletal abnormalities and lethality [161].

#### 2.2.6. Other Genes Recently Implicated in TA

Mutations in *GREM2*, which encodes GREMLIN2, were identified in 7 patients with TA (hypodontia) and additional malformations, including taurodontism, sparse and slow-growing hair and dry and itchy skin [132]. GREMLIN2 is known to regulate BMPs in embryonic development. Specifically, *BMP4* has an important role in tooth development and its knockdown resulted in the arrest of tooth development in mice [8]. Interestingly, *GREM2* knockout mice have small and malformed teeth but do not have tooth development arrested. However, these findings suggest a potential role for *GREM2* during tooth development [162]. Three missense mutations in *GREM2* (p.Ala13Val, p.Glu136Asp and p.Gln76Glu) were identified as pathogenic in individuals with isolated TA and have not been reported in association with other structural malformations. *GREM2* mutations exhibit variable expressivity even within the same families [132].

*EDA*, *EDAR* and *EDARADD* have also been suggested to contribute to isolated TA [98,129,163]. In a genotype–phenotype correlation study, all EDA mutations in individuals with isolated TA were missense mutations and most likely to be located in the TNF domain [163].

Another WNT pathway gene, *WNT10B* (wingless-type MMTV integration site family, member 10B)*,* has also been implicated in isolated TA, albeit mostly in families from China and Thailand. Three heterozygous missense mutations (c.632G > A, p.Arg211Gln; c.569C > G, p.Pro190Arg; and c.851T > G, p.Phe284Cys) and one nonsense mutation (c.786G > A, p.Trp262*) in *WNT10B* were identified in Chinese individuals with TA, especially those missing the upper lateral incisors [151]. More recently, two additional heterozygous missense mutations (c.475G > C, p.Ala159Pro and c.1052G > A, p.Arg351His) were identified in five Thai families, and associated with isolated TA and other dental anomalies including microdontia and taurodontism [150].

A homozygous missense variant c.1312C > T (p.Arg438Cys) in *ANTXR1* (anthrax toxin receptor 1) was identified in association with TA (oligodontia) in a Turkish family [10]. Homozygous and biallelic variants in *ANTXR1* have been associated with Growth retardation, Alopecia, Pseudoanodontia, and Optic atrophy (GAPO) syndrome, characterized by delayed growth, alopecia, failure of tooth eruption, and optic atrophy segregating as an AR trait [18,19,164]. Targeted disruption of *Antxr1* in mice resulted in viable mice without major structural defects, although dental overgrowth, incisor misalignment, and dental dysplasia were observed, due to an accumulation of extracellular matrix in various tissues [165]. Antxr1 expression was detected in the epithelium of developing tongue, maxillary and mandibular processes, as well as in the dental epithelium and mesenchyme at early stages of tooth development. At later stages, Antxr1 expression was noted in the epithelium of the enamel organ and in the dental papilla, and then shifted to the polarized layer of ameloblasts and differentiating odontoblasts. *ANTXR1* is a tumor-specific endothelial marker implicated in colorectal cancer, and upregulated in tumor angiogenesis [166,167,168]. Previously, Lammi et al. [22] showed that variants in the tumor suppressor gene *AXIN2* segregated in a family with severe TA (oligodontia) and colorectal cancer, and suggested that TA and colorectal cancer may have a common genetic etiology. Numerous studies have since reported on variations in cancer-related genes in association with TA [123,169,170]. These findings highlight the complex nature of TA and emphasize the need to consider modifier genes and/or gene-gene interactions in studies of this condition.

A recent genome-wide association study (GWAS) that included over 1900 TA cases and 330,000 controls of European ancestry identified 4 novel risk variants that associate with TA, and 5 that associate with a combined phenotype of TA plus oral-facial clefts [171]. Dental anomalies are frequent findings in children with oral-facial clefts, and cleft subphenotypes have been proposed based on the pattern of the associated dental anomalies [172]. Of the 9 variants found, 5 were located in or close to Wnt pathway genes that have been implicated in tooth development and/or development of other ectodermal structures (*EDA*, *EDAR*, *FOXI3*, *FORXP1* and *LEF1*), and 4 were located in or close to genes that have not been implicated in TA or tooth development (*ASCL5/CACNA1S, ARHGAP15, NOL11* and *FAM49A*). In addition, two known variants in *WNT10A* (p.Phe228Ile and p.Cys107*) were also found to be significantly associated with TA in this GWAS [171].

## 3. Monogenic vs. Oligogenic Inheritance Models

In recent years, oligogenic inheritance and multi-locus variation models have been proposed for a number of Mendelian diseases, further establishing the concept of mutational load in human genetic disease [173]. For TA, evidence for oligogenic inheritance is emerging, supported by the findings of recent whole exome sequencing studies and/or direct sequencing studies with more than one candidate gene [10,11].

Digenic mutations in *MSX1* and *PAX9* had been reported as associated with a more severe TA phenotype (15–17 missing teeth) [142], and the interactions between these genes had begun to be elucidated. Studies have shown that *PAX9* interacts with *MSX1* to synergistically activate the expression of downstream tooth development genes, i.e., *BMP4*, which is essential for proper tooth morphogenesis [2]. The presence of digenic mutations in these genes might abolish their interactions and thus lead to more severe TA phenotypes [142].

More recently, biallelic or heterozygous genotypes of *WNT10A* were found in TA patients who also presented homozygous or heterozygous genotypes of *EDA*, *EDAR* or *EDARADD*, suggesting the combined phenotypic effects of alleles in distinct genes as contributing to TA [174]. Additionally, compound heterozygous mutations in *WNT10A* (IVS2 + 1G > A and c.637G > A) were identified segregating together with a missense heterozygous variant in *GREM2* (c.38C > T) in a patient with TA of maxillary permanent canines [132].

Additional heterozygous splicing mutations in *DKK1* (dickkopf WNT signaling pathway inhibitor 1; c.548-4G > T) and in *COL17A1* (collagen type XVII alpha 1 chain; c.3277 + 3G > C), and a heterozygous missense variant in *LAMA3* (laminin subunit alpha 3; c.2798G > T) were identified segregating with TA in one consanguineous Turkish family [11]. Pathogenic mutations in these genes had not yet been identified in individuals with TA, although they can be considered biologically plausible candidate genes due to their biological roles and/or disease-associated phenotypes. *DKK1* encodes a high-affinity dickkopf homolog 1 transmembrane receptor that cooperates with *LRP6* to block Wnt signaling during development and other cellular processes [175]. In mice, *DKK1* is expressed in the dental mesenchyme, odontoblasts and osteoblasts, and its ectopic expression in the oral epithelia of transgenic mouse embryos resulted in blocked epithelial and mesenchymal signaling leading to arrest of tooth development at the early bud stage [176]. A common single-nucleotide polymorphism in *DKK1* (rs11001553) was previously associated with isolated TA in a Chinese Han population [126].

Mutations in *LAMA3* cause junctional epidermolysis bullosa (OMIM #226650), an autosomal recessive skin disorder characterized by the presence of multiple blisters and erosions, dystrophic nails, enamel hypoplasia and hypodontia [177]. Further, targeted disruption of *LAMA3* in mice resulted in defects of ameloblast differentiation [178]. Variations in *COL17A1* have also been described in epidermolysis bullosa patients with enamel defects [179].

A homozygous variant (c.-387delC > G) in the 5’ UTR of the *PITX2* gene, described above as etiologic for Axenfeld–Rieger syndrome, and a homozygous missense variant in *BMP4* (c.T455C, p.Val152Ala) were identified segregating with isolated TA in two siblings from an Italian family [180].

The finding of likely pathogenic alleles in more than one locus suggests the potential for oligogenic inheritance and multilocus variation models in isolated TA, likely contributing to the variable phenotypes. With advances in genome-wide sequencing studies of well-characterized TA individuals and families, and careful genotype-phenotype correlations, new TA genes acting individually or interactively with other genes are likely to be identified.

## 4. Genetic Pathways as the Focus of Future Studies

Over the years, studies using transgenic animals demonstrated that defects in genes belonging to BMP, FGF and WNT signaling pathways resulted in severe abnormalities of tooth development ranging from defects in tooth patterning to complete arrest of tooth development [1,4,8]. Meanwhile, mutations in genes belonging to the FGF family have not yet been described in association with TA, whereas a single variant in *BMP4* (see above) was found in one TA family.

The current available evidence supports a significant role for WNT pathway genes in isolated TA, mostly supported by the higher frequency of pathogenic mutations in *AXIN2*, *WNT10A*, *WNT10B* and *LRP6* in TA individuals [11,22,150,171]. WNT signaling molecules are essential for patterning, proliferation and differentiation of multiple cell types during embryonic development. Secretion of WNTs, particularly *WNT4*, *WNT6* and *WNT10*, from the dental epithelium has been reported as critical for tooth development, as the absence of WNT signaling leads to a dysfunctional enamel knot and subsequently in arrest of tooth development [103,181].

*EDA*, *EDAR* and *EDARADD*, with roles in both syndromic and isolated TA, belong to the NF-kB signaling pathway. Additional genes in the NF-kB pathway include *NEMO* (inhibitor of nuclear factor kappa B kinase subunit gamma), an important pathway modulator, and *TRAF6* (TNF receptor associated factor 6), although little is known about the exact roles of these genes in tooth development and variations in these two genes were reported in individuals with syndromic TA [4].

**Figure 2:**
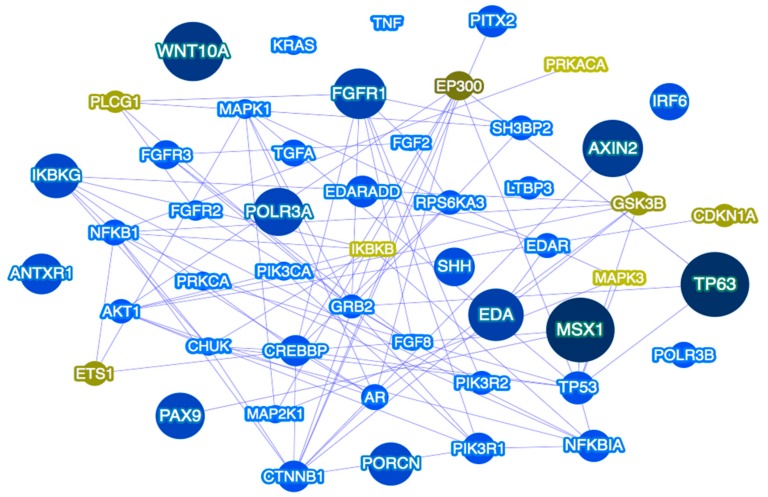
Tooth agenesis gene network as predicted by Phenolyzer [183]. The network shown includes the top 50 prioritized genes, and their predicted relations with seed genes. Larger dark blue nodes indicate seed genes, medium royal blue nodes indicate interacting genes. Green font indicates predicted genes. Blue lines indicate protein–protein interactions.

Based on the aforementioned observations of multilocus variation as a potential explanation for some TA cases, with candidate genes belonging to the same or different signaling pathways, it is presumable to hypothesize that isolated TA may be the result of variation in more than one gene, acting individually or in combination with other genes and contributing to the variable expressivity of the condition [182]. Determining the full spectrum of putative defective genes in TA, the pathways in which they belong (Figure 2), their functions and interactive partners, will allow for improving our understanding of the underlying mechanisms in TA and may be the basis of future prevention and tooth replacement strategies.

## 5. Conclusions

Isolated TA is a heterogeneous condition with variable expressivity. While variations in numerous genes have been attributed as causal for TA, the etiology of TA in many individuals is still unsolved and may reflect mutations in genes yet unknown to tooth development, or the presence of multilocus variation. Moreover, environmental and epigenetic factors may also be considered likely contributors to TA phenotypes and should be explored in future studies. Next-generation sequencing studies of well-characterized individuals and families present the unique ability to identify all of the TA-predisposing variants throughout the genome while revealing important genetic and network interactions that may be critical for tooth development. Further genetic and functional studies focusing on newly identified genes and pathways have the potential to elucidate the genetic landscape of isolated TA and provide insights into preventive and treatment strategies. Targeted therapeutics for TA-relevant genes and/or pathways may represent future tooth replacement therapies.

## Figures and Tables

**Table 1:** Genes involved in syndromic tooth agenesis and associated phenotypes.

Gene/Locus	OMIM	Chromosome	Syndrome	Inheritance	Dental/Oral Phenotypes	Animal Model	Animal Model Phenotype	Reference
*ADAMTS2*	604539	5q35.3	Ehlers–Danlos syndrome	AR	Hypodontia, microdontia, tooth discoloration	Yes Bovine	Dermatosparatic phenotype resembling EDS type VII C	[15,16,17]
*ANTXR1*	606410	2p13.3	Growth retardation, alopecia, pseudoanodontia, and optic atrophy (GAPO) syndrome	AR	Hypodontia, delayed eruption	Yes	Growth delay, bone loss, shortened skulls with frontal bossing, and midfacial hypoplasia	[18,19,20]
*AXIN2*	604025	17q24.1	Oligodontia-colorectal cancer syndrome	AD	Oligodontia	Yes	Abnormal cranium morphology	[21,22]
*COL1A1/2*	120150	17q21.33	Osteogenesis imperfecta type 1	AD	Hypodontia, oligodontia	Yes	Lethal, bone fractures	[23]
*CREBBP*	600140	16p13.3	Rubinstein–Taybi syndrome	AD	Hypodontia, retrognathia, micrognathia, arched/narrow palate, talon cusps, dental crowding, screwdriver incisors, cross bite, and enamel hypoplasia	Yes	Skeletal malformations	[24]
*EDA*	300451	Xq13.1	Ectodermal dysplasia, hypohidrotic	XLR	Anodontia, hypodontia, misshapen teeth, microdontia	Yes Canine	Incomplete set of conically shaped teeth	[25,26,27,28]
*EDAR*	604095	2q13	Ectodermal dysplasia, hypohidrotic/hair/tooth type	AR	Anodontia, hypodontia, oligodontia	Yes Mouse	Decreased molar number, small incisor, small molars, abnormal enamel knot morphology	[29,30,31,32,33]
*EDARADD*	606603	1q42-q43	Ectodermal dysplasia, hypohidrotic/hair/tooth type	AD	Anodontia, hypodontia, taurodontism, microdontia	Yes Mouse	Abnormal tooth morphology, decreased molar number, small molars, abnormal enamel morphology	[34,35,36]
*EVC*	604831	4p16.2	Ellis–van Creveld syndrome and Weyers acrofacial dysostosis	AR/AD	Natal teeth, enamel abnormalities, hypodontia, microdontia	Yes Mouse	Enamel defects, abnormal tooth morphology	[37,38,39]
*EVC2*	607261	4p16.2	Ellis–van Creveld syndrome and Weyers acrofacial dysostosis	AR/AD	Natal teeth, enamel abnormalities, hypodontia, oligodontia, microdontia	Yes Mouse	Microdontia, small upper incisors, small cranium	[37,38,39,40]
*FGF10*	602115	5p12	Lacrimoauriculodentodigital syndrome	AD	Hypodontia (maxillary incisors), microdontia, delayed eruption, enamel dysplasia	Yes Mouse	Abnormal tooth morphology, short incisors, small molars, abnormal palatal development, abnormal tongue morphology	[41,42,43]
*FGFR1*	136350	8p11.23	Kallmann syndrome	XLR	Hypodontia, cleft lip/palate	Yes Mouse	Abnormal cranium morphology, facial asymmetry, long incisors	[44]
*FGFR2*	176943	10q26.13	Lacrimoauriculodentodigital syndrome	AD	Hypodontia (maxillary incisors), microdontia peg laterals, delayed eruption, enamel dysplasia	Yes Mouse	Arrest of tooth development, long incisors, decreased molar number, micrognathia	[43]
			Apert syndrome	AD	Hypodontia (maxillary canines), enamel opacities, ectopic eruptions, gingival hyperplasia	Yes Mouse	Arrest of tooth development, long incisors, decreased molar number, micrognathia	[45,46,47]
*FGFR3*	134934	4p16.3	Crouzon syndrome with acanthosis nigricans	AD	Hypodontia, malocclusion, cementomas, delayed eruption, midface hypoplasia	Yes Mouse	Tooth misalignment, long incisors, malocclusion, prognathia, maxillary retrognathia	[48,49,50]
*FLNB*	603381	3p14.3	Larsen syndrome	AD	Hypodontia, delayed dental development, class 3 occlusion, morphological anomalies	Yes Mouse	Abnormal cranium morphology	[51]
*FOXC1*	601090	6p25.3	Axenfeld–Rieger syndrome type 3	AD	Hypodontia, microdontia, taurodontism	Yes, Mouse	Short mandible	[52,53]
*GJA1*	121014	6q22.31	Oculodentodigital dysplasia	AD, AR	Microdontia, enamel hypoplasia, hypodontia, delayed eruption	Yes Mouse	Abnormal tooth morphology, microdontia, small mandible and maxilla, reduced enamel thickness	[54,55]
*GRHL2*	608576	8q22.3	Ectodermal dysplasia/short stature syndrome	AR	Delayed eruption, hypodontia, enamel hypoplasia	Yes, Mouse	Abnormal cranium morphology, facial and midline clefts	[56]
*IRF6*	607199	1q32.2	van der Woude syndrome	AD	Hypodontia, cleft lip/palate	Yes Mouse	Abnormal tooth morphology, abnormal palatal development, small mandible	[57,58]
*JAG1*	601920	20p12.2	Alagille Syndrome	AD	Hypodontia, enamel hypoplasia and opacities, hypomineralization	Yes mouse	Short maxilla, malocclusion, abnormal palate morphology	[59]
*KDM6A*	300128	Xp11.3	Kabuki syndrome 2	XLD	High-arched palate, malocclusion, microdontia, a small dental arch, hypodontia, severe maxillary recession, conical teeth	Yes Mouse	Cranioschisis	[60,61]
*KMT2D*	602113	12q13.12	Kabuki syndrome 1	AD	High-arched palate, malocclusion, microdontia, a small dental arch, hypodontia, severe maxillary retrognathia, conical teeth	Yes Mouse	Short maxilla, flattened snout	[62,63]
*KREMEN1*	609898	22q12.1	Ectodermal dysplasia, hair/tooth type	AR	Oligodontia, hypodontia, alveolar ridge deficiency, increased palatal depth	Yes	No craniofacial phenotype	[64]
*MKKS*	604896	20p12.2	Bardet-Biedl syndrome	AR	Dental crowding, high-arched palate, hypodontia, malocclusion, enamel hypoplasia, retrognathia	Yes Mouse	Abnormal olfactory epithelium	[65]
*MSX1*	142983	4p16.1	Witkop syndrome	AD	Hypodontia, oligodontia	Yes Mouse	Arrest of tooth development, nail bed and nail plates defective, cleft palate	[66]
*NEMO*	300248	Xq28	Incontinentia pigmenti	XLD	Hypodontia, anodontia, microdontia	Yes Mouse	No craniofacial phenotype	[67,68]
*NSD1*	606681	5q35.3	Sotos syndrome I	AD	Hypodontia, enamel defects, malocclusion	Yes Mouse	No craniofacial phenotypes	[69,70]
*OFD1*	300170	Xp22.2	Orofaciodigital syndrome I	XLD	Hypodontia, missing lateral incisors, canine malposition, micrognathia	Yes	Primary cilia formed then disappeared, renal cysts	[71,72]
*P63*	603273	3q28	Orofacial cleft 8, Rapp-Hodgkin, and Ectrodactyly, ectodermal dysplasia, and cleft lip/palate syndrome 3	AD	Hypodontia, enamel hypoplasia, extensive dental caries, hypodontia of the mandibular canines, generalized microdontia, prominent marginal ridges of permanent maxillary incisors, round-shaped permanent molars, and barrel-shaped permanent maxillary central incisors	Yes Mouse	Arrest of tooth development, small mandible and maxilla, abnormal craniofacial development, cleft palate	[73,74,75]
*PITX2*	601542	4q25	Axenfeld–Rieger syndrome, type 1	AD	Hypodontia, microdontia, enamel hypoplasia	Yes Mouse	Abnormal maxilla and mandible morphology, arrested tooth development	[53,76,77,78]
*PVRL1*	600644	11q23.3	Cleft lip/palate-ectodermal dysplasia	AR	Hypodontia, cleft lip and palate, abnormal dental morphology, microdontia	Yes Mouse	Abnormal tooth morphology	[79,80]
*RECQL4*	603780	8q24.3	Rothmund–Thomson syndrome	AR	Hypodontia, microdontia, hypoplastic teeth	Yes Mouse	Delayed tooth eruption, cleft palate	[81]
*RSK2*	300075	Xp22.12	Coffin–Lowry syndrome	XLD	High narrow palate, midline lingual furrow, hypodontia, and microdontia	Yes Mouse	Abnormal tooth morphology, supernumerary teeth	[82]
*SHH*	600725	7q36.3	Holoprosencephaly	AD	Cleft lip and palate, single central incisor, micrognathia	Yes	Abnormal tooth morphology, microdontia	[83,84]
*TBX3*	601621	12q24.21	Ulnar-mammary syndrome	AD	Hypodontia, ectopic and hypoplastic canines	Yes	Secondary palate clefting	[85]
*TCOF1*	606847	5q32-q33	Treacher Collins syndrome	AD	Hypodontia, micrognathia, malocclusion, spaced teeth	Yes	Short mandible and maxilla	[86,87]
*TFAP2B*	601601	6p12.3	Char syndrome	AD	Oligodontia, hypodontia, thick lips, retention of primary teeth	Yes	No craniofacial phenotype	[88]
*Trisomy 21*	190685	21q22.13	Down syndrome	IC	Hypodontia, delayed eruption, barrel-shaped permanent maxillary central incisors	Yes	General hypoplasia and developmental delay, hydronephrosis, heart and neurologic defects	[89,90]
*TSPEAR*	612920	21q22.3	Ectodermal dysplasia	AR	Hypodontia, microdontia	Yes	No craniofacial phenotypes	[91]
*UBR1*	605981	15q15.2	Johanson–Blizzard syndrome	AR	Oligodontia	Yes	No craniofacial phenotypes	[92]
*WNT10A*	606268	2q35	Odontoonychodermal dysplasia	AR	Oligodontia, hypodontia, microdontia	Yes	Arrested tooth development of molars, supernumerary molars, abnormal tooth morphology	[93,94,95]
			Schopf–Schulz–Passarge syndrome	AR	Oligodontia, hypodontia, microdontia	Yes	Arrested tooth development of molars, supernumerary molars, abnormal tooth morphology	[94,96,97]

OMIM, Online Mendelian Inheritance in Man (https://www.ncbi.nlm.nih.gov/omim/) AR, autosomal recessive; AD, autosomal dominant, XLD, x-linked dominant, XLR, x-linked recessive; IC, isolated cases.

**Table 2:** Genes involved in isolated tooth agenesis.

Gene	OMIM	Chromosome	Dental Phenotypes	Reference(s)
*AXIN2*	604025	17q24.1	Oligodontia, hypodontia	[22,98,124,125]
*ANTXR1*	606410	2p13.3	Oligodontia, hypodontia	[10]
*COL17A1*	113811	10q25.1	Hypodontia	[11]
*DKK1*	605189	10q21.1	Hypodontia	[11,126]
*EDA*	300451	Xq13.1	Oligodontia, hypodontia	[33,127,128,129,130]
*EDAR*	604095	2q13	Oligodontia, hypodontia	[33]
*EDARADD*	606603	1q42-q43	Oligodontia, hypodontia	[33]
*FGFR1*	136350	8p11.23	Hypodontia	[131]
*GREM2*	608832	1q43	Hypodontia, microdontia, taurodontia	[132]
*IRF6*	607199	1q32.2	Hypodontia, lip pits	[131,133]
*MSX1*	142983	4p16.2	Oligodontia, hypodontia	[98,134,135]
*LAMA3*	600805	18q11.2	Hypodontia	[11]
*LRP6*	603507	12p13.2	Oligodontia	[11,136,137]
*LTBP3*	602090	11q13.1	Oligodontia, hypodontia	[138,139]
*PAX9*	167416	14q13.3	Oligodontia, hypodontia, microdontia	[98,140,141,142,143,144,145]
*SMOC2*	607223	6q27	Oligodontia, microdontia, abnormal morphology	[146,147]
*WNT10A*	606268	2q35	Oligodontia, hypodontia	[11,108,109,110,148,149]
*WNT10B*	601906	12q13.12	Oligodontia, microdontia	[150,151]

## References

[1] Bei M. (2009). Molecular genetics of tooth development. Curr. Opin. Genet. Dev..

[2] Thesleff I., Vaahtokari A., Vainio S., Jowett A. (1996). Molecular mechanisms of cell and tissue interactions during early tooth development. Anat. Rec..

[3] Gorlin R.J., Cohen M.M., Levin S.L. (1990). Syndromes of the Head and Neck.

[4] Yin W., Bian Z. (2015). The gene network underlying hypodontia. J. Dent. Res..

[5] Polder B.J., Van’t Hof M.A., Van der Linden F.P., Kuijpers-Jagtman A.M. (2004). A meta-analysis of the prevalence of dental agenesis of permanent teeth. Commun. Dent. Oral Epidemiol..

[6] Carter K., Worthington S. (2015). Morphologic and demographic predictors of third molar agenesis: A systematic review and meta-analysis. J. Dent. Res..

[7] Ye X., Attaie A.B. (2016). genetic basis of nonsyndromic and syndromic tooth agenesis. J. Pediatr. Genet..

[8] Lan Y., Jia S., Jiang R. (2014). Molecular patterning of the mammalian dentition. Semin. Cell Dev. Biol..

[9] Wang J., Sun K., Shen Y., Xu Y., Xie J., Huang R., Zhang Y., Xu C., Zhang X., Wang R. (2016). DNA methylation is critical for tooth agenesis: Implications for sporadic non-syndromic anodontia and hypodontia. Sci. Rep..

[10] Dinckan N., Du R., Akdemir Z.C., Bayram Y., Jhangiani S.N., Doddapaneni H., Hu J., Muzny D.M., Guven Y., Aktoren O. (2018). A biallelic *ANTXR1* variant expands the anthrax toxin receptor associated phenotype to tooth agenesis. Am. J. Med. Genet. A.

[11] Dinckan N., Du R., Petty L.E., Coban-Akdemir Z., Jhangiani S.N., Paine I., Baugh E.H., Erdem A.P., Kayserili H., Doddapaneni H. (2018). Whole-exome sequencing identifies novel variants for tooth agenesis. J. Dent. Res..

[12] Nieminen P. (2009). Genetic basis of tooth agenesis. J. Exp. Zool. B Mol. Dev. Evol..

[13] Howe B.J., Cooper M.E., Vieira A.R., Weinberg S.M., Resick J.M., Nidey N.L., Wehby G.L., Marazita M.L., Moreno Uribe L.M. (2015). Spectrum of dental phenotypes in nonsyndromic orofacial clefting. J. Dent. Res..

[14] Vieira A.R. (2003). Oral clefts and syndromic forms of tooth agenesis as models for genetics of isolated tooth agenesis. J. Dent. Res..

[15] Colige A., Nuytinick L., Hausser I., van Esse A.J., Thiry M., Herens C., Adès L.C., Malfait F., Paepe A.D., Franck P. (2004). Novel types of mutation responsible for the dermatosparactic type of Ehlers-Danlos syndrome (type VIIC) and common polymorphisms in the *ADAMTS2* gene. J. Invest. Dermatol..

[16] Colige A., Sieron A.L., Li S.W., Schwarze U., Petty E., Wertelecki W., Wilcox W., Krakow D., Cohn D.H., Reardon W. (1999). Human Ehlers-Danlos syndrome type VIIC and bovine dermatosparaxis are caused by mutations in the procollagen I N-proteinase gene. Am. J. Hum. Genet..

[17] Bekhouche M., Colige A. (2015). The procollagen N-proteinases *ADAMTS2*, *3* and *14* in pathophysiology. Matrix Biol..

[18] Bayram Y., Pehlivan D., Karaca E., Gambin T., Jhangiani S.N., Erdin S., Elcioglu N.H. (2014). Whole exome sequencing identifies three novel mutations in *ANTXR1* in families with GAPO syndrome. Am. J. Med. Genet. A.

[19] Stranecky V., Hoischen A., Hartmannová H., Zaki M.S., Chaudhary A., Zudaie E., Nosková L., Baresová V., Pristoupilová A., Hodanová K. (2013). Mutations in *ANTXR1* cause GAPO syndrome. Am. J. Hum. Genet..

[20] Salas-Alanis J.C., Scott C.A., Fajardo-Ramírez O.R., Duran C., Moreno-Treviño M.G., Kelsell D.P. (2016). New *ANTXR1* gene mutation for GAPO syndrome: A case report. Mol. Syndromol..

[21] Marvin M.L., Mazzoni S.M., Herron C.M., Edwards S., Gruber S.B., Petty E.M. (2011). *AXIN2*-associated autosomal dominant ectodermal dysplasia and neoplastic syndrome. Am. J. Med. Genet. A.

[22] Lammi L., Arte S., Somer M., Järvinen H., Lahermo P., Thesleff I., Pirinen S., Nieminen P. (2004). Mutations in *AXIN2* cause familial tooth agenesis and predispose to colorectal cancer. Am. J. Hum. Genet..

[23] Malmgren B., Andersson K., Lindahl K., Kindmark A., Grigelioniene G., Zachariadis V., Dahllöf G., Aström E. (2017). Tooth agenesis in osteogenesis imperfecta related to mutations in the collagen type I genes. Oral Dis..

[24] Bloch-Zupan A., Stachtou J., Emmanouil D., Arveiler B., Griffiths D., Lacombe D. (2007). Oro-dental features as useful diagnostic tool in Rubinstein-Taybi syndrome. Am. J. Med. Genet. A.

[25] Monreal A.W., Zonana J., Ferguson B. (1998). Identification of a new splice form of the *EDA1* gene permits detection of nearly all X-linked hypohidrotic ectodermal dysplasia mutations. Am. J. Hum. Genet..

[26] Han D., Gong Y., Wu H., Zhang X., Yan M., Wang X., Qu H., Feng H., Song S. (2008). Novel *EDA* mutation resulting in X-linked non-syndromic hypodontia and the pattern of *EDA*-associated isolated tooth agenesis. Eur. J. Med. Genet..

[27] Lexner M.O., Bardow A., Juncker I., Jensen L.G., Almer L., Kreiborg S., Hertz J.M. (2008). X-linked hypohidrotic ectodermal dysplasia. Genetic and dental findings in 67 Danish patients from 19 families. Clin. Genet..

[28] Tao R., Jin B., Guo S.Z., Qing W., Feng G.Y., Brooks D.G., Liu L., Xu J., Li T., Yan Y. (2006). A novel missense mutation of the *EDA* gene in a Mongolian family with congenital hypodontia. J. Hum. Genet..

[29] Henningsen E., Svendsen M.T., Lidballe D.L., Jensen P.K. (2014). A novel mutation in the *EDAR* gene causes severe autosomal recessive hypohidrotic ectodermal dysplasia. Am. J. Med. Genet. A.

[30] Naeem M., Muhammad D., Ahmad W. (2005). Novel mutations in the *EDAR* gene in two Pakistani consanguineous families with autosomal recessive hypohidrotic ectodermal dysplasia. Br. J. Dermatol..

[31] Naqvi S.K., Wasif N., Javaid H., Ahmad W. (2011). Two novel mutations in the gene *EDAR* causing autosomal recessive hypohidrotic ectodermal dysplasia. Orthod. Craniofac. Res..

[32] Shimomura Y., Sato N., Miyashita A., Hashimoto T., Ito M., Kuwano R. (2004). A rare case of hypohidrotic ectodermal dysplasia caused by compound heterozygous mutations in the *EDAR* gene. J. Invest. Dermatol..

[33] Zeng B., Zhao Q., Li S., Lu H., Lu J., Ma L., Zhao W., Yu D. (2017). Novel *EDA* or *EDAR* Mutations identified in patients with X-linked hypohidrotic ectodermal dysplasia or non-syndromic tooth agenesis. Genes.

[34] Bal E., Baala L., Cluzeau C., El Kerch F., Ouldim K., Hadj-Rabia S., Bodemer C., Munnich A., Courtois G., Sefiani A. (2007). Autosomal dominant anhidrotic ectodermal dysplasias at the EDARADD locus. Hum. Mutat..

[35] Headon D.J., Emmal S.A., Ferguson B.M., Tucker A.S., Justice M.J., Sharpe P.T., Zonana J., Overbeek P.A. (2001). Gene defect in ectodermal dysplasia implicates a death domain adapter in development. Nature.

[36] Wohlfart S., Söder S., Smahi A., Schneider H. (2016). A novel missense mutation in the gene *EDARADD* associated with an unusual phenotype of hypohidrotic ectodermal dysplasia. Am. J. Med. Genet. A.

[37] Ruiz-Perez V.L., Ide S.E., Strom T.M., Lorenz B., Wilson D., Woods K., King L., Francomano C., Freisinger P., Spranger S. (2000). Mutations in a new gene in Ellis-van Creveld syndrome and Weyers acrodental dysostosis. Nat. Genet..

[38] D’Asdia M.C., Torrent I., Consoli F., Ferese R., Magliozzi M., Bernardini L., Guida V., Diglio M.C., Marino B., Dallapiccola B. (2013). Novel and recurrent *EVC* and *EVC2* mutations in Ellis-van Creveld syndrome and Weyers acrofacial dyostosis. Eur. J. Med. Genet..

[39] Ye X., Song G., Fan M., Shi L., Jabs E.W., Huang S., Guo R., Bian Z. (2006). A novel heterozygous deletion in the *EVC2* gene causes Weyers acrofacial dysostosis. Hum Genet.

[40] Shen W., Han D., Zhang J., Zhao H., Feng H. (2011). Two novel heterozygous mutations of *EVC2* cause a mild phenotype of Ellis-van Creveld syndrome in a Chinese family. Am. J. Med. Genet. A.

[41] Entesarian M., Matsson H., Klar J., Bergendal B., Olson L., Arakaki R., Hayashi Y., Ohuchi H., Falahat B., Bolstad A.I. (2005). Mutations in the gene encoding fibroblast growth factor 10 are associated with aplasia of lacrimal and salivary glands. Nat Genet.

[42] Milunsky J.M., Zhao G., Maher T.A., Colby R., Everman D.B. (2006). LADD syndrome is caused by *FGF10* mutations. Clin. Genet..

[43] Rohmann E., Brunner H.G., Kayserili H., Uyguner O., Nürnberg G., Lew E.D., Dobbie A., Eswarakumar V.P., Uzumcu A., Ulubil-Emeroglu A. (2006). Mutations in different components of *FGF* signaling in LADD syndrome. Nat. Genet..

[44] Bailleul-Forestier I., Gros C., Zenaty D., Bennaceur S., Leger J., de Roux N. (2010). Dental agenesis in Kallmann syndrome individuals with *FGFR1* mutations. Int. J. Paediatr. Dent..

[45] Letra A., de Almeida A.L., Kaizer R., Esper L.A., Sgarbosa S., Granjeiro J.M. (2007). Intraoral features of Apert’s syndrome. Oral Surg. Oral Med. Oral Pathol. Oral Radiol. Endod..

[46] Stavropoulos D., Bartzela T., Bronkhorst E., Mohlin B., Hagberg C. (2011). Dental agenesis patterns of permanent teeth in Apert syndrome. Eur. J. Oral Sci..

[47] Ibrahimi O.A., Chiu E.S., McCarthy J.G., Mohammadi M. (2005). Understanding the molecular basis of Apert syndrome. Plast. Reconstr. Surg..

[48] Meyers G.A., Orlow S.J., Munro I.R., Przylepa K.A., Jabs E.W. (1995). Fibroblast growth factor receptor 3 (*FGFR3*) transmembrane mutation in Crouzon syndrome with acanthosis nigricans. Nat. Genet..

[49] Reardon W., Wilkes D., Rutland P., Pulleyn L.J., Malcolm S., Dean J.C., Evans R.D., Jones B.M., Hayward R., Hall C.M. (1997). Craniosynostosis associated with *FGFR3 pro250arg* mutation results in a range of clinical presentations including unisutural sporadic craniosynostosis. J. Med. Genet..

[50] Wilkes D., Rutland P., Pulleyn L.J., Reardon W., Moss C., Ellis J.P., Winter R.M., Malcolm S. (1996). A recurrent mutation, ala391glu, in the transmembrane region of *FGFR3* causes Crouzon syndrome and acanthosis nigricans. J. Med. Genet..

[51] Tsang M.C., Ling J.Y., King N.M., Chow S.K. (1986). Oral and craniofacial morphology of a patient with Larsen syndrome. J. Craniofac. Genet. Dev. Biol..

[52] Berenstein-Aizman G., Hazan-Molina H., Drori D., Aizenbud D. (2011). Axenfeld-Rieger syndrome: Dentofacial manifestation and oral rehabilitation considerations. Pediatr. Dent..

[53] O’Dwyer E.M., Jones D.C. (2005). Dental anomalies in Axenfeld-Rieger syndrome. Int. J. Paediatr. Dent..

[54] Kjaer K.W., Hansen L., Eiberg H., Leicht P., Opitz J.N., Tommerup N. (2004). Novel *Connexin 43* (*GJA1*) mutation causes oculo-dento-digital dysplasia with curly hair. Am. J. Med. Genet. A.

[55] Paznekas W.A., Karczeski B., Vermeer S., Lowry R.B., Delatycki M., Laurence F., Koivisto P.A., Van Maldergem L., Boyadjiev S.A., Bodurtha J.N. (2009). *GJA1* mutations, variants, and connexin 43 dysfunction as it relates to the oculodentodigital dysplasia phenotype. Hum. Mutat..

[56] Petrof G., Nanda A., Howden J., Takeichi T., McMillan J.R., Aristodemou S., Ozoemena L., Liu L., South A.P., Purreyron C. (2014). Mutations in *GRHL2* result in an autosomal-recessive ectodermal Dysplasia syndrome. Am. J. Hum. Genet..

[57] Kondo S., Schutte B.C., Richardsson R.J., Bjork B.C., Knight A.S., Watanabe Y., Howard E., de Lima R.L., Daack-Hirsch S., Sander A. (2002). Mutations in *IRF6* cause Van der Woude and popliteal pterygium syndromes. Nat. Genet..

[58] Zucchero T.M., Cooper M.E., Maher B.S., Daak-Hirsch S., Nepomuceno B., Ribeiro L., Caprau D., Christensen K., Suzuki Y., Machida J. (2004). Interferon regulatory factor 6 (*IRF6*) gene variants and the risk of isolated cleft lip or palate. N. Engl. J. Med..

[59] Ho N.C., Lacbawan F., Francomano C.A., Ho V. (2000). Severe hypodontia and oral xanthomas in Alagille syndrome. Am. J. Med. Genet..

[60] Lederer D., Shears D., Benoit V., Verellen-Dumoulin C., Maystadt I. (2014). A three generation X-linked family with Kabuki syndrome phenotype and a frameshift mutation in *KDM6A*. Am. J. Med. Genet. A.

[61] Van Laarhoven P.M., Neitzel L.R., Quintana A.M., Geiger E.A., Zackai E.H., Clouthier D.E., Artinger K.B., Ming J.E., Shaik T.H. (2015). Kabuki syndrome genes *KMT2D* and *KDM6A*: Functional analyses demonstrate critical roles in craniofacial, heart and brain development. Hum. Mol. Genet..

[62] Lerone M., Priolo M., Naselli A., Vignolo A., Romeo G., Silengo M.C. (1997). Ectodermal abnormalities in Kabuki syndrome. Am. J. Med. Genet..

[63] Matsune K., Matsune K., Shimizu T., Tohma T., Asada Y., Ohashi H., Maeda T. (2001). Craniofacial and dental characteristics of Kabuki syndrome. Am. J. Med. Genet..

[64] Issa Y.A., Kamal L., Rayyan A.A., Dweik D., Pierce S., Lee M.K., King M.C., Walsh T., Kanaan M. (2016). Mutation of *KREMEN1*, a modulator of *Wnt* signaling, is responsible for ectodermal dysplasia including oligodontia in Palestinian families. Eur. J. Hum. Genet..

[65] Forsythe E., Kenny J., Bacchelli C., Beales P.L. (2018). Managing Bardet-Biedl syndrome-now and in the future. Front Pediatr..

[66] Jumlongras D., Bei M., Stimson J.M., Wen-Fang W., DePalma S.R., Seidman C.E., Felbor U., Maas R., Seidman J.G. (2001). A nonsense mutation in *MSX1* causes Witkop syndrome. Am. J. Hum. Genet..

[67] Aradhya S., Woffendin H., Jakins T., Bardaro T., Esposito T., Smahi A., Shaw C., Levy M., Munnich A., D’Urso M. (2001). A recurrent deletion in the ubiquitously expressed *NEMO* (*IKK*-*gamma*) gene accounts for the vast majority of incontinentia pigmenti mutations. Hum. Mol. Genet..

[68] Ku C.L., Dupuis-Girod S., Dittrich A.M., Bustamante J., Santos O.F., Schulze I., Bertrand Y., Couly G., Bodemer C., Bossuy X. (2005). *NEMO* mutations in 2 unrelated boys with severe infections and conical teeth. Pediatrics.

[69] Hirai N., Matsune K., Ohashi H. (2011). Craniofacial and oral features of Sotos syndrome: Differences in patients with submicroscopic deletion and mutation of *NSD1* gene. Am. J. Med. Genet. A.

[70] Kotilainen J., Pohjola P., Pirinen S., Arte S., Niminen P. (2009). Premolar hypodontia is a common feature in Sotos syndrome with a mutation in the *NSD1* gene. Am. J. Med. Genet. A.

[71] Shotelersuk V., Tifft C.J., Vacha S., Peters K.F., Biesecker L.G. (1999). Discordance of oral-facial-digital syndrome type 1 in monozygotic twin girls. Am. J. Med. Genet..

[72] Larralde de Luna M., Raspa M.L., Ibargoyen J. (1992). Oral-facial-digital type 1 syndrome of Papillon-Leage and Psaume. Pediatr. Dermatol..

[73] Brunner H.G., Hamel B.C., Bokhoven H.V. (2002). *P63* gene mutations and human developmental syndromes. Am. J. Med. Genet..

[74] Rodini E.O., Freitas J.A., Richieri-Costa A. (1990). Rapp-Hodgkin syndrome: Report of a Brazilian family. Am. J. Med. Genet..

[75] Sripathomsawat W., Tanpaiboon P., Heering J., Dötsch V., Hennekam R.C., Kantaputra P. (2011). Phenotypic analysis of *Arg227* mutations of *TP63* with emphasis on dental phenotype and micturition difficulties in EEC syndrome. Am. J. Med. Genet. A.

[76] Brooks B.P., Moroi S.E., Downs C.A., Wiltse S., Othman M.I., Semina E.V., Richards J.E. (2004). A novel mutation in the *PITX2* gene in a family with Axenfeld-Rieger syndrome. Ophthalmic Genet..

[77] Lu M.F., Pressman C., Dyer R., Johnson R.L., Martin J.F. (1999). Function of Rieger syndrome gene in left-right asymmetry and craniofacial development. Nature.

[78] Li X., Venugopalan S.R., Cao H., Pinho F.O., Paine M.L., Snead M.L., Semina E.V., Amendt B.A. (2014). A model for the molecular underpinnings of tooth defects in Axenfeld-Rieger syndrome. Hum. Mol. Genet..

[79] Bustos T., Simosa V., Pinto-Cisternas J., Abramovits W., Jolay L., Rodriguez L., Fernandez L., Ramela M. (1991). Autosomal recessive ectodermal dysplasia: I. An undescribed dysplasia/malformation syndrome. Am. J. Med. Genet..

[80] Sozen M.A., Suzuki K., Tolarova M.M., Bustos T., Fernandez Iglesias J.E., Spritz R.A. (2001). Mutation of *PVRL1* is associated with sporadic, non-syndromic cleft lip/palate in northern Venezuela. Nat. Genet..

[81] Starr D.G., McClure J.P., Connor J.M. (1985). Non-dermatological complications and genetic aspects of the Rothmund-Thomson syndrome. Clin. Genet..

[82] Hunter A.G. (2002). Coffin-Lowry syndrome: A 20-year follow-up and review of long-term outcomes. Am. J. Med. Genet..

[83] Nanni L., Ming J.E., Du Y., Hall R.K., Aldred M., Bankier A., Muenke M. (2001). *SHH* mutation is associated with solitary median maxillary central incisor: A study of 13 patients and review of the literature. Am. J. Med. Genet..

[84] Petryk A., Graf D., Marcucio R. (2015). Holoprosencephaly: Signaling interactions between the brain and the face, the environment and the genes, and the phenotypic variability in animal models and humans. Wiley Interdiscip. Rev. Dev. Biol..

[85] Bamshad M., Le T., Watjkins W.S., Dixon M.E., Kramer B.E., Roeder A.D., Carey J.C., Root S., Schinzel A., Van Maldergem L. (1999). The spectrum of mutations in *TBX3*: Genotype/Phenotype relationship in ulnar-mammary syndrome. Am. J. Hum. Genet..

[86] Lowry R.B., Morgan K., Holmes T.M., Metcalf P.J., Stauffer G.F. (1985). Mandibulofacial dysostosis in Hutterite sibs: A possible recessive trait. Am. J. Med. Genet..

[87] Li C., Mernagh J., Bourgeois J. (2009). Novel craniofacial and extracraniofacial findings in a case of Treacher Collins syndrome with a pathogenic mutation and a missense variant in the *TCOF1* gene. Clin. Dysmorphol..

[88] Mani A., Radhakrishnan J., Farhi A., Carew K.S., Waarnes C.A., Nelson-Williams C., Day R.W., Pober B., State M.W., Lifton R.P. (2005). Syndromic patent ductus arteriosus: Evidence for haploinsufficient *TFAP2B* mutations and identification of a linked sleep disorder. Proc. Natl. Acad. Sci. USA.

[89] Korenberg J.R. (1993). Toward a molecular understanding of Down syndrome. Prog. Clin. Biol. Res..

[90] Palaska P.K., Antonarakis G.S. (2016). Prevalence and patterns of permanent tooth agenesis in individuals with Down syndrome: A meta-analysis. Eur. J. Oral Sci..

[91] Peled A., Sarig O., Samuelov L., Bertolini M., Ziv L., Weissglas-Volkov D., Eskin-Schwartz M., Adase C.A., Malchin N., Bochner R. (2016). Mutations in *TSPEAR*, encoding a regulator of notch signaling, affect tooth and hair follicle morphogenesis. PLoS Genet..

[92] Almashraki N., Abdulnabee M.Z., Sukalo M., Alrajoudi A., Sharafadeen I., Zenker M. (2011). Johanson-Blizzard syndrome. World J. Gastroenterol..

[93] Adaimy L., Chouery E., Megarbane H., Mroueh S., Delague V., Nicolas E., Belguith H., de Mazancourt P., Megarbane A. (2007). Mutation in *WNT10A* is associated with an autosomal recessive ectodermal dysplasia: The odonto-onycho-dermal dysplasia. Am. J. Hum. Genet..

[94] Bohring A., Stamm T., Spaich C., Haase C., Spree K., Hehr U., Hoffmann M., Ledig S., Sel S., Wieacker P. (2009). *WNT10A* mutations are a frequent cause of a broad spectrum of ectodermal dysplasias with sex-biased manifestation pattern in heterozygotes. Am. J. Hum. Genet..

[95] Nawaz S., Klar J., Wajid M., Aslam M., Tariq M., Schuster J., Baig S.M., Dahl N. (2009). *WNT10A* missense mutation associated with a complete odonto-onycho-dermal dysplasia syndrome. Eur. J. Hum. Genet..

[96] Monk B.E., Pieris S., Soni V. (1992). Schopf-Schulz-Passarge syndrome. Br. J. Dermatol..

[97] Schopf E., Schulz H.J., Passarge E. (1971). Syndrome of cystic eyelids, palmo-plantar keratosis, hypodontia and hypotrichosis as a possible autosomal recessive trait. Birth Defects Orig. Artic. Ser..

[98] Bergendal B., Klar J., Stecksén-Blicks C., Norderyd J., Dahl N. (2011). Isolated oligodontia associated with mutations in *EDARADD*, *AXIN2*, *MSX1*, and *PAX9* genes. Am. J. Med. Genet. A.

[99] Hudson C.D., Witkop C.J. (1975). Autosomal dominant hypodontia with nail dysgenesis. Report of twenty-nine cases in six families. Oral Surg. Oral Med. Oral Pathol..

[100] Satokata I., Maas R. (1994). *Msx1* deficient mice exhibit cleft palate and abnormalities of craniofacial and tooth development. Nat. Genet..

[101] Jarvinen E., Salazar-Ciudad I., Birchmeier W., Taketo M.M., Jernvall J., Thesleff I. (2006). Continuous tooth generation in mouse is induced by activated epithelial *Wnt/beta*-catenin signaling. Proc. Natl. Acad. Sci. USA.

[102] Liu F., Millar S.E. (2010). *Wnt/beta*-catenin signaling in oral tissue development and disease. J. Dent. Res..

[103] Yamashiro T., Zheng L., Shitaku Y., Saito M., Tsubakimoto T., Takada K., Takano-Yamamoto T., Thesleff I. (2007). *Wnt10a* regulates dentin sialophosphoprotein *mRNA* expression and possibly links odontoblast differentiation and tooth morphogenesis. Differentiation.

[104] Chen J., Lany Y., Baek J.A., Gao Y., Jiang R. (2009). *Wnt/beta*-catenin signaling plays an essential role in activation of odontogenic mesenchyme during early tooth development. Dev. Biol..

[105] Yang J., Wang S.K., Choi M., Reid B.M., Hu Y., Lee Y.L., Herzog C.R., Kim-Berman H., Lee M., Benke P.J. (2015). Taurodontism, variations in tooth number, and misshapened crowns in *Wnt10a* null mice and human kindreds. Mol. Genet. Genomic Med..

[106] Cluzeau C., Hadj-Rabia S., Jambou M., Mansour S., Guigue P., Masmoudi S., Bal E., Chassaing N., Vincent M.C., Viot G. (2011). Only four genes (*EDA1*, *EDAR*, *EDARADD*, and *WNT10A*) account for 90% of hypohidrotic/anhidrotic ectodermal dysplasia cases. Hum. Mutat..

[107] Plaisancie J., Bailleul-Forestier I., Gaston V., Vaysse F., Lacombe D., Holder-Espinasse M., Abramowicz M., Coubes C., Plessis G., Faivre L. (2013). Mutations in *WNT10A* are frequently involved in oligodontia associated with minor signs of ectodermal dysplasia. Am. J. Med. Genet. A.

[108] Song S., Zhao R., He H., Zhang J., Feng H., Lin L. (2014). *WNT10A* variants are associated with non-syndromic tooth agenesis in the general population. Hum. Genet..

[109] van den Boogaard M.J., Créton M., Bronkhorst Y., van der Hout A., Hennekam E., Lindhout D., Cune M., van Amstel H.K.P. (2012). Mutations in *WNT10A* are present in more than half of isolated hypodontia cases. J. Med. Genet..

[110] Kantaputra P., Sripathomsawat W. (2011). *WNT10A* and isolated hypodontia. Am. J. Med. Genet. A.

[111] Ellwanger K., Saito H., Clément-Lacroix P., Maltry N., Niedermeyer J., Lee W.K., Baron R., Rawadi G., Westphal H., Niehrs C. (2008). Targeted disruption of the Wnt regulator Kremen induces limb defects and high bone density. Mol. Cell Biol..

[112] Ingraham C.R., Kinoshita A., Kondo S., Yang B., Sajan S., Trout K.J., Malik M.I., Dunnwald M., Goudy S.L., Lovett M. (2006). Abnormal skin, limb and craniofacial morphogenesis in mice deficient for interferon regulatory factor 6 (Irf6). Nat. Genet..

[113] Leslie E.J., Mancuso J.L., Schutte B.C., Cooper M.E., Durda K.M., L’heureux J., Zucchero T.M., Marazita M.L., Murray J.C. (2013). Search for genetic modifiers of IRF6 and genotype-phenotype correlations in Van der Woude and popliteal pterygium syndromes. Am. J. Med. Genet. A.

[114] Ludwig K.U., Mangold E., Herms S., Nowak S., Reutter H., Paul A., Becker J., Herberz R., AlChawa T., Nasser E., Böhmer A.C. (2012). Genome-wide meta-analyses of nonsyndromic cleft lip with or without cleft palate identify six new risk loci. Nat. Genet..

[115] Fitch N., Kaback M. (1978). The Axenfeld syndrome and the Rieger syndrome. J. Med. Genet..

[116] Chisholm I.A., Chudley A.E. (1983). Autosomal dominant iridogoniodysgenesis with associated somatic anomalies: Four-generation family with Rieger’s syndrome. Br. J. Ophthalmol..

[117] Semina E.V., Reiter R., Leysens N.J., Alward W.L.M., Small K.W., Datson N.A., Siegel-Bartelt J., Bierke-Nelson D., Bitoun P., Zabel B.U. (1996). Cloning and characterization of a novel bicoid-related homeobox transcription factor gene, *RIEG*, involved in Rieger syndrome. Nat. Genet..

[118] Liu W., Selever J., Lu M.F., Martin J.F. (2003). Genetic dissection of *Pitx2* in craniofacial development uncovers new functions in branchial arch morphogenesis, late aspects of tooth morphogenesis and cell migration. Development.

[119] Hjalt T.A., Semina E.V., Amendt B.A., Murray J.C. (2000). The Pitx2 protein in mouse development. Dev. Dyn..

[120] Gardner E.J. (1962). Follow-up study of a family group exhibiting dominant inheritance for a syndrome including intestinal polyps, osteomas, fibromas and epidermal cysts. Am. J. Hum. Genet..

[121] Letra A., Bjork B., Cooper M.E., Szabo-Rogers H., Deleyiannis F.W.B., Field L.L., Czeizel A.E., Ma L., Garlet G.P., Poletta F.A. (2012). Association of *AXIN2* with non-syndromic oral clefts in multiple populations. J. Dent. Res..

[122] Lindor N.M., Win A.K., Gallinger S., Daftary D., Thibodeau S.N., Silva R., Letra A. (2014). Colorectal cancer and self-reported tooth agenesis. Hered. Cancer Clin. Pract..

[123] Williams M.A., Biguetti C., Romero-Bustillos M., Maheshwari K., Dinckan N., Cavalla F., Vieira A.R. (2018). Colorectal cancer-associated genes are associated with tooth agenesis and may have a role in tooth development. Sci. Rep..

[124] Callahan N., Modesto A., Meira R., Seymen F., Patir A., Vieira A.R. (2009). Axis inhibition protein 2 (AXIN2) polymorphisms and tooth agenesis. Arch. Oral Biol..

[125] Mostowska A., Biedziak B., Jagodzinski P.P. (2006). Axis inhibition protein 2 (AXIN2) polymorphisms may be a risk factor for selective tooth agenesis. J. Hum. Genet..

[126] Liu H.C., Zhang J., Wong S., Han D., Zhao H.S., Feng H.L. (2014). Association between rs11001553 of DKK1 and non-syndromic tooth agenesis in the Chinese Han population. Genet. Mol. Res..

[127] Mues G., Tardivel A., Willen L., Kapadia H., Seaman R., Frazier-Bowers S., Schneider P., D’Souza R.N. (2010). Functional analysis of Ectodysplasin-A mutations causing selective tooth agenesis. Eur. J. Hum. Genet..

[128] Shen W., Wang Y., Liu Y., Liu H., Zhao H., Zhang G., Snead M.L., Han D., Feng H. (2016). Functional study of ectodysplasin-a mutations causing non-syndromic tooth agenesis. PLoS ONE.

[129] Song S., Han D., Qu H., Gong Y., Wu H., Zhang X., Zhong N., Feng H. (2009). *EDA* gene mutations underlie non-syndromic oligodontia. J. Dent. Res..

[130] Sarkar T., Bansal R., Das P. (2014). Whole genome sequencing reveals novel non-synonymous mutation in *ectodysplasin A* (*EDA*) associated with non-syndromic X-linked dominant congenital tooth agenesis. PLoS ONE.

[131] Vieira A.R., Modesto A., Meira R., Barbosa A.R., Lidral A.C., Murray J.C. (2007). Interferon regulatory factor 6 (*IRF6*) and fibroblast growth factor receptor 1 (*FGFR1*) contribute to human tooth agenesis. Am. J. Med. Genet. A.

[132] Kantaputra P.N., Kaewgahya M., Hatsadaloi A., Vogel P., Kawasaki K., Ohazama A., Ketudat Cairns J.R. (2015). *GREMLIN 2* mutations and dental anomalies. J. Dent. Res..

[133] Vieira A.R., Seymen F., Patir A., Menezes R. (2008). Evidence of linkage disequilibrium between polymorphisms at the *IRF6* locus and isolate tooth agenesis, in a Turkish population. Arch. Oral Biol..

[134] Lidral A.C., Reising B.C. (2002). The role of *MSX1* in human tooth agenesis. J. Dent. Res..

[135] Vastardis H., Karimbux N., Guthua S.W., Seidman J.G., Seidman C.E. (1996). A human *MSX1* homeodomain missense mutation causes selective tooth agenesis. Nat. Genet..

[136] Massink M.P., Créton M.A., Spanevello F., Fennis W.M., Cune M.S., Savelberg S.M., Nijman I.J., Maurice M.M., van den Boogaard M.J.H., van Haaften G. (2015). Loss-of-function mutations in the WNT co-receptor *LRP6* cause autosomal-dominant oligodontia. Am. J. Hum. Genet..

[137] Ockeloen C.W., Khandelwal K.D., Dreesen K., Ludwig K.U., Sullivan R., Van Rooij I.A., Thonissen M., Swinnen S., Phan M., Conte F. (2016). Novel mutations in *LRP6* highlight the role of WNT signaling in tooth agenesis. Genet. Med..

[138] Dugan S.L., Temme R.T., Olson R.A., Mikhailov A., Law R., Mahmood H., Noor A., Vincent J.B. (2015). New recessive truncating mutation in *LTBP3* in a family with oligodontia, short stature, and mitral valve prolapse. Am. J. Med. Genet. A.

[139] Noor A., Windpassinger C., Vitcu I., Orlic M., Rafiq M.A., Khalid M., Malik M.N., Ayub M., Alman B., Vincent J.B. (2009). Oligodontia is caused by mutation in *LTBP3*, the gene encoding latent *TGF-beta* binding protein 3. Am. J. Hum. Genet..

[140] Sarkar T., Bansal R., Das P. (2017). A novel G to A transition at initiation codon and exon-intron boundary of *PAX9* identified in association with familial isolated oligodontia. Gene.

[141] Vieira A.R., Meira R., Modesto A., Murray J.C. (2004). *MSX1*, *PAX9*, and *TGFA* contribute to tooth agenesis in humans. J. Dent. Res..

[142] Wong S.W., Han D., Zhang H., Liu Y., Zhang X., Miao M.Z., Wang Y., Zhao N., Zeng L., Bai B. (2018). Nine novel *PAX9* mutations and a distinct tooth agenesis genotype-phenotype. J. Dent. Res..

[143] Frazier-Bowers S.A., Guo D.C., Cavender A., Xue L., Evans B., King T., Milewicz D., D’Souza R.N. (2002). A novel mutation in human *PAX9* causes molar oligodontia. J. Dent. Res..

[144] Mitsui S.N., Yasue A., Masuda K., Watanabe K., Horiuchi S., Imopto I., Tanaka E. (2014). Novel *PAX9* mutations cause non-syndromic tooth agenesis. J. Dent. Res..

[145] Stockton D.W., Das P., Goldenberg M., D’Souza R.N., Patel P.I. (2000). Mutation of *PAX9* is associated with oligodontia. Nat. Genet..

[146] Alfawaz S., Fong F., Plagnol V., Wong F.S., Fearne J., Kelsell D.P. (2013). Recessive oligodontia linked to a homozygous loss-of-function mutation in the *SMOC2* gene. Arch. Oral Biol..

[147] Bloch-Zupan A., Jamet X., Etard C., Lauget V., Muller J., Geoffroy V., Straus J.P., Pelletier V., Marion V., Poch O. (2011). Homozygosity mapping and candidate prioritization identify mutations, missed by whole-exome sequencing, in *SMOC2*, causing major dental developmental defects. Am. J. Hum. Genet..

[148] Arzoo P.S., Klar J., Bergendal B., Norderyd J., Dahl N. (2014). *WNT10A* mutations account for (1/4) of population-based isolated oligodontia and show phenotypic correlations. Am. J. Med. Genet. A.

[149] Yuan Q., Zhao M., Tandon B., Maili L., Liu X., Zhang A., Baugh E.H., Tran T., Silva R.M., Hecht J.T. (2017). Role of *WNT10A* in failure of tooth development in humans and zebrafish. Mol. Genet. Genomic. Med..

[150] Kantaputra P.N., Hutsadaloi A., Kaewgahya M., Intachai W., German R., Koparal M., Leethanakul C., Tolun A., Ketudat Cairns J.R. (2018). *WNT10B* mutations associated with isolated dental anomalies. Clin. Genet..

[151] Yu P., Yang W., Han D., Wang X., Guo S., Li J., Li F., Zhang X., Wong S.W., Bai B. (2016). Mutations in *WNT10B* are identified in individuals with oligodontia. Am. J. Hum. Genet..

[152] Vastardis H. (2000). The genetics of human tooth agenesis: New discoveries for understanding dental anomalies. Am. J. Orthod. Dentofacial Orthop..

[153] Liang J., Von den Hoff J., Lange J., Ren Y., Bian Z., Carels C.E. (2016). *MSX1* mutations and associated disease phenotypes: Genotype-phenotype relations. Eur. J. Hum. Genet..

[154] Peters H., Neubüser A., Kratochwil K., Balling R. (1998). *Pax9*-deficient mice lack pharyngeal pouch derivatives and teeth and exhibit craniofacial and limb abnormalities. Genes Dev..

[155] Peters H., Neubuser A., Balling R. (1998). Pax genes and organogenesis: *Pax9* meets tooth development. Eur. J. Oral. Sci..

[156] Bonczek O., Balcar V.J., Sery O. (2017). *PAX9* gene mutations and tooth agenesis: A review. Clin. Genet..

[157] Brook A.H., Elcock C., Aggarwal M., Lath D.L., Russell J.M., Patel P.I., Smith R.N. (2009). Tooth dimensions in hypodontia with a known *PAX9* mutation. Arch. Oral Biol..

[158] Wang J., Jian F., Chen J., Wang H., Lin Y., Yang Z., Pan X., Lai W. (2011). Sequence analysis of *PAX9*, *MSX1* and *AXIN2* genes in a Chinese oligodontia family. Arch. Oral Biol..

[159] Vink C.P., Ockeloen C.W., Ten Kate S., Koolen D.A., Van Amstel J.K.P., Kuijpers-Jagtman A.M., Van Heumen C.C., Kleefstra T., Carels C.E. (2014). Variability in dentofacial phenotypes in four families with *WNT10A* mutations. Eur. J. Hum. Genet..

[160] He H., Han D., Feng H., Qu H., Song S., Bai B., Zhang Z. (2013). Involvement of and interaction between *WNT10A* and *EDA* mutations in tooth agenesis cases in the Chinese population. PLoS ONE.

[161] Pinson K.I., Brennan J., Monkley S., Avery B.J., Skarnes W.C. (2000). An LDL-receptor-related protein mediates Wnt signalling in mice. Nature.

[162] Vogel P., Liu J., Platt K.A., Read R.W., Thiel M., Vance R.B., Brommage R. (2015). Malformation of incisor teeth in *Grem2*−/− mice. Vet. Pathol..

[163] Zhang J., Han D., Song S., Wang Y., Zhao H., Pan S., Bai B., Feng H. (2011). Correlation between the phenotypes and genotypes of X-linked hypohidrotic ectodermal dysplasia and non-syndromic hypodontia caused by *ectodysplasin-A* mutations. Eur. J. Med. Genet..

[164] Tipton R.E., Gorlin R.J. (1984). Growth retardation, alopecia, pseudo-anodontia, and optic atrophy—The GAPO syndrome: Report of a patient and review of the literature. Am. J. Med. Genet..

[165] Cullen M., Seaman S., Chaudhary A., Yang M.Y., Hilton M.B., Logsdon D., Croix B.S. (2009). Host-derived tumor endothelial marker 8 promotes the growth of melanoma. Cancer Res..

[166] Carson-Walter E.B., Watkins D.N., Nanda A., Vogelstein B., Kinzler K.W., Croix B.S. (2001). Cell surface tumor endothelial markers are conserved in mice and humans. Cancer Res..

[167] Fernando S., Fletcher B.S. (2009). Targeting tumor endothelial marker 8 in the tumor vasculature of colorectal carcinomas in mice. Cancer Res..

[168] Liu P., Xie G., Geng P., Zheng C., Li J., Pan F., Liang H. (2015). Anti-tumor angiogenesis effect of genetic fusion vaccine encoding murine β-defensin 2 and tumor endothelial marker-8 in a CT-26 murine colorectal carcinoma model. Int. J. Clin. Exp. Med..

[169] Yue H., Liang J., Yang K., Hua B., Bian Z. (2016). Functional analysis of a novel missense mutation in *AXIN2* associated with non-syndromic tooth agenesis. Eur. J. Oral Sci..

[170] Yamaguchi T., Hosomichi K., Yano K., Kim Y.I., Nakaoka H., Kimura R., Shirota T. (2017). Comprehensive genetic exploration of selective tooth agenesis of mandibular incisors by exome sequencing. Hum. Genome Var..

[171] Jonsson L., Magnusson T.E., Thordarson A., Jonsson T., Geller F., Feenstra B., Rivadeneira F. (2018). Rare and common variants conferring risk of tooth agenesis. J. Dent. Res..

[172] Letra A., Menezes R., Granjeiro J.M., Vieira A.R. (2007). Defining subphenotypes for oral clefts based on dental development. J. Dent. Res..

[173] Posey J.E., Harel T., Liu P., Rosenfeld J.A., James R.A., Coban Akdemir Z.H., Xia F. (2017). Resolution of disease phenotypes resulting from multilocus genomic variation. N. Engl. J. Med..

[174] Arte S., Parmanen S., Pirinen S., Alaluusua S., Nieminen P. (2013). Candidate gene analysis of tooth agenesis identifies novel mutations in six genes and suggests significant role for WNT and EDA signaling and allele combinations. PLoS ONE.

[175] Fedi P., Bafico A., Soria A.N., Burgess W.H., Miki T., Bottaro D.P., Aaronson S.A. (1999). Isolation and biochemical characterization of the human Dkk-1 homologue, a novel inhibitor of mammalian Wnt signaling. J. Biol. Chem..

[176] Li J., Huang X., Xu X., Mayo J., Bringas P. Jr., Jiang R., Wang S., Chai Y. (2011). *SMAD4*-mediated WNT signaling controls the fate of cranial neural crest cells during tooth morphogenesis. Development.

[177] McGrath J.A., Kivirikko S., Ciatti S., Moss C., Dunnill M.G.S., Eady R.A., Uitto J. (1995). A homozygous nonsense mutation in the α3 chain gene of laminin 5 (*LAMA3*) in Herlitz junctional epidermolysis bullosa: Prenatal exclusion in a fetus at risk. Genomics.

[178] Ryan M.C., Lee K., Miyashita Y., Carter W.G. (1999). Targeted disruption of the *LAMA3* gene in mice reveals abnormalities in survival and late stage differentiation of epithelial cells. J. Cell Biol..

[179] Tasanen K., Eble J.A., Aumailley M., Schumann H., Baetge J., Tu H., Bruckner-Tuderman L. (2000). Collagen XVII is destabilized by a glycine substitution mutation in the cell adhesion domain Col15. J. Biol. Chem..

[180] Salvi A., Giacopuzzi E., Bardellini E., Amadori F., Ferrari L., De Petro G., Majorana A. (2016). Mutation analysis by direct and whole exome sequencing in familial and sporadic tooth agenesis. Int. J. Mol. Med..

[181] Zhu X., Zhao P., Liu Y., Zhang X., Fu J., Yu H.M.I., Zhang Z. (2013). Intra-epithelial requirement of canonical Wnt signaling for tooth morphogenesis. J. Biol. Chem..

[182] De Coster P.J., Marks L.A., Martens L.C., Huysseune A. (2009). Dental agenesis: Genetic and clinical perspectives. J. Oral Pathol. Med..

[183] Phenolyzer. http://phenolyzer.wglab.org.

